# Knockout of VDAC1 in H9c2 Cells Promotes Oxidative Stress-Induced Cell Apoptosis through Decreased Mitochondrial Hexokinase II Binding and Enhanced Glycolytic Stress

**DOI:** 10.33594/000000274

**Published:** 2020-09-09

**Authors:** Meiying Yang, Jie Sun, David F. Stowe, Emad Tajkhorshid, Wai-Meng Kwok, Amadou K. S. Camara

**Affiliations:** a Department of Anesthesiology, Medical College of Wisconsin, Milwaukee, WI, USA,; b Institute of Clinical Medicine Research, Department of Gastroenterology and Hepatology, Suzhou Hospital affiliated with Nanjing Medical University, Suzhou, Jiangsu, China,; c Research Service, Zablocki VA Medical Center, Milwaukee, WI, USA,; d Department of Physiology, Medical College of Wisconsin, Milwaukee, WI, USA,; e Cardiovascular Center, Medical College of Wisconsin, Milwaukee, WI, USA,; f NIH Center for Macromolecular Modeling and Bioinformatics, Beckman Institute for Advanced Science and Technology, University of Illinois at Urbana Champaign, Urbana, IL, USA,; g Department of Biochemistry, and Center for Biophysics and Quantitative Biology, University of Illinois at Urbana Champaign, Urbana, IL, USA,; h Cancer Center, Medical College of Wisconsin, Milwaukee, WI, USA,; i Department of Pharmacology and Toxicology, Medical College of Wisconsin, Milwaukee, WI, USA

**Keywords:** VDAC1 knockout, Oxidative stress, Mitochondria-bound hexokinase II, Extracellular acidification, Cell death/apoptosis, Bax

## Abstract

**Background/Aims:**

The role of VDAC1, the most abundant mitochondrial outer membrane protein, in cell death depends on cell types and stimuli. Both silencing and upregulation of VDAC1 in various type of cancer cell lines can stimulate apoptosis. In contrast, in mouse embryonic stem (MES) cells and mouse embryonic fibroblasts (MEFs), the roles of VDAC1 knockout (VDAC1^−/−^) in apoptotic cell death are contradictory. The contribution and underlying mechanism of VDAC1^−/−^ in oxidative stress-induced cell death in cardiac cells has not been established. We hypothesized that VDAC1 is an essential regulator of oxidative stress-induced cell death in H9c2 cells.

**Methods:**

We knocked out VDAC1 in this rat cardiomyoblast cell line with CRISPR-Cas9 genome editing technique to produce VDAC1^−/−^ H9c2 cells, and determined if VDAC1 is critical in promoting cell death via oxidative stress induced by tert-butylhydroper-oxide (tBHP), an organic peroxide, or rotenone (ROT), an inhibitor of mitochondrial complex I by measuring cell viability with MTT assay, cell death with TUNEL stain and LDH release. The mitochondrial and glycolytic stress were examined by measuring O_2_ consumption rate (OCR) and extracellular acidification rate (ECAR) with a Seahorse XFp analyzer.

**Results:**

We found that under control conditions, VDAC1^−/−^ did not affect H9c2 cell proliferation or mitochondrial respiration. However, compared to the wildtype (WT) cells, exposure to either tBHP or ROT enhanced the production of ROS, ECAR, and the proton (H^+^) production rate (PPR) from glycolysis, as well as promoted apoptotic cell death in VDAC1^−/−^ H9c2 cells. VDAC1^−/−^ H9c2 cells also exhibited markedly reduced mitochondria-bound hexokinase II (HKII) and Bax. Restoration of VDAC1 in VDAC1^−/−^ H9c2 cells reinstated mitochondria-bound HKII and concomitantly decreased tBHP and ROT-induced ROS production and cell death. Interestingly, mitochondrial respiration remained the same after tBHP treatment in VDAC1^−/−^ and WT H9c2 cells.

**Conclusion:**

Our results suggest that VDAC1^−/−^ in H9c2 cells enhances oxidative stress-mediated cell apoptosis that is directly linked to the reduction of mitochondria-bound HKII and concomitantly associated with enhanced ROS production, ECAR, and PPR.

## Introduction

The voltage dependent anion channel (VDAC), the most abundant protein in the outer mitochondrial membrane (OMM), is a multifunctional protein that is involved in the transport of ions, e.g. Ca^2+^, and metabolites/substrates, e.g. ADP/ATP, between the mitochondrial intermembrane space (IMS] and the cytosol. In this role, VDAC is a key factor in mediating cellular energy production for cell survival over apoptosis. It alleviates apoptosis in part by binding with hexokinase II (HKII], which prevents pro-apoptotic proteins, e.g. Bax/Bak, from binding to the OMM [1, 2]. VDAC is also a key channel for maintaining a close anatomical and functional association with the sarcoplasmic reticulum [2]. Of the three isoforms of VDAC, VDACs 1, 2 and 3 found in mammals, VDAC1 is the most abundant in cardiac mitochondria, where it plays a substantial role in cardiac ischemia-reperfusion (IR) injury [3–6].

The mechanism of VDAC1 in modulating cell death/survival in the setting of cardiac IR injury is not well understood. In prior reports, we found that cardiac IR injury induced VDAC1 tyrosine nitration, an irreversible and deleterious post-translational modification [7]. Tyrosine nitration of VDAC1 alters its interaction with adenine nucleotide translocase (ANT) and HKII in mitochondria [8]. We observed further that nitration of VDAC1 increased oligomerization and channel conductance that was accompanied by compromised cardiac function on reperfusion [7, 8].

Knockout of VDAC1 (VDAC1^−/−^) has been examined in mouse embryonic stem (MES) cells, mice, and mouse embryonic fibroblasts (MEFs). In MES cells, VDAC1^−/−^ reduced the basal O_2_ consumption and uncoupled O_2_ consumption induced by CCCP; however, respiratory control ratios and cell growth rates remained unchanged compared to wildtype (WT) [9]. The authors concluded that VDAC1 is not required for cell viability in this model. In VDAC1^−/−^ mice, there were also abnormalities in mitochondrial respiration in both cardiac and skeletal muscle and the mitochondrial ultrastructure was altered, but the mice remained viable [9–13]. Mitochondria isolated from VDAC1^−/−^ and WT mouse hearts exhibited similar changes in Ca^2+^ induced swelling and cytochrome *c* release, suggesting no differences in mitochondrial permeability transition pore (mPTP) opening caused by Ca^2+^ overload and/or oxidative stress [10]. These studies suggested that VDAC1 is not essential for formation of the mPTP and that VDAC1 does not contribute directly to apoptosis via this mechanism.

Other reports assessed effects of VDAC1^−/−^ on cell death using VDAC1^−/−^ MEFs, but the conclusions from these studies are contradictory. Brahimi-Horn et al. [14] reported that VDAC1^−/−^ MEFs were more sensitive to apoptotic and chemotherapeutic agents, such as staurosporine and bleomycin, compared to WT MEFs. In contrast, Baines et al. [10] showed that cell death of VDAC1^−/−^ MEFs and WT MEFs induced by ionomycin, H_2_O_2_ and classic apoptotic agents, for examples, Bax overexpression and staurosporine, were comparable between the two groups.

Because VDAC1 plays crucial roles in mitochondrial function, cell survival and apoptosis, it is perhaps not unexpected that knockout of VDAC1 has differing outcomes depending on cell types and stress conditions. It may also seem counterintuitive that the impact of VDAC1 knock out leads only limited deficiencies in cellular function in some cells. Since mitochondrial function is a critical determinant of cardiac cell function, we investigated the mechanistic role of VDAC1 during oxidative stress-induced cell death, specifically in a cardiac cell line. We hypothesized that VDAC1 plays a pivotal role in modulating oxidative stress-induced cell death in cardiac cells. To test this, we knocked out VDAC1 in H9c2 (VDAC1^−/−^ H9c2) cells, a rat cardiomyoblast cell line, and investigated the impact on oxidative stress induced by tert-butylhydroperoxide (tBHP), an organic peroxide, or rotenone (ROT), an inhibitor of mitochondrial complex I, compared to VDAC1 WT, as an underlying mechanism in the induction of cell death. We found that VDAC1^−/−^ H9c2 cells were more sensitive to tBHP- and ROT-induced cell death, compared to WT, because of reduced mitochondria-bound HKII, enhanced generation of ROS, and augmented extracellular acidification rate (ECAR] and proton (H^+^) production rate (PPR) due to increased lactate and H^+^ production by glycolysis.

## Materials and Methods

### Cell culture and cell transfection

H9c2 cells were cultured in Dulbecco’s modified Eagle’s medium (DMEM), supplemented with 10% fetal bovine serum (FBS) under 95% air, 5% CO_2_ at 37°C, and sub-cultured when at 50–60% confluence. For restoration of VDAC1 in VDAC1 knockout cells, a stable cell line expressing VDAC1 in VDAC1^−/−^ H9c2 cells was generated. In brief, rat VDAC1 full-length cDNA (GenBank: BC072484, purchased from Open Biosystems) was cloned into the expression vector pcDNA3.1 vector (Invitrogen) to make VDAC1 construct. The VDAC1 construct was then transfected into VDAC1^−/−^ H9c2 cells by the Neon Transfection system. The VDAC1 transfected cells were selected by adding G418 (1 mg/ml) into media for 14 days and then the G418-resis-tant cells, i.e. VDAC1 expression VDAC1^−/−^ H9c2 cells, were expanded in 500 μg/ml G418.

### Generation of VDAC1^−/−^ H9c2 cells using CR1SPR/Cas9 genome editing

**Fig. 1.**
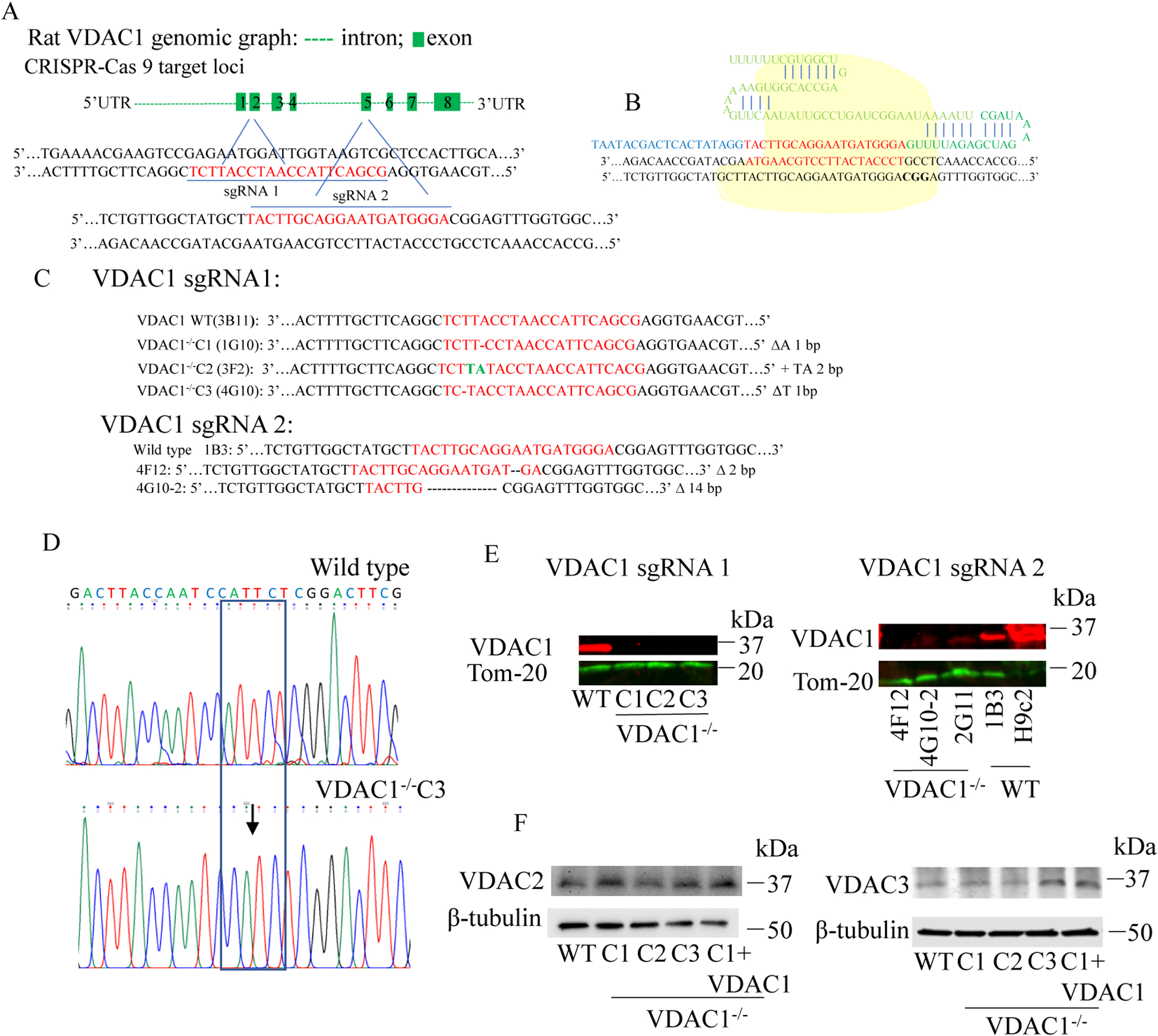
Generation and identification of VDACl^−/−^ H9c2 cells. (A) Rat VDACl genomic graph and the sites and sequences of two single guide (sg) RNAs in the rat genome. (B) Diagram of complex formation of sgRNA, T7 promoter, CRISPR guide RNA and Cas9 protein. (C) The gene mutation sequences in selected VDAC1^−/−^ H9c2 cell clones from sgRNA1 and 2. (D) Alignment of DNA sequencing traces of VDAC1^−/−^C3 to WT H9c2 cells. Arrow in the box indicates a single nucleotide T deletion in the VDAC1^−/−^C3 H9c2 cell clone. (E) Western blot analyses of selected WT and VDAC1^−/−^ H9c2 cell clones with VDAC1 specific and Tom-20 (loading control) antibodies. (F) Western blot analyses of selected WT and VDAC1^−/−^ H9c2 cell clones with VDAC2, VDAC3 and β-tubulin (loading control) antibodies.

To generate VDAC1^−/−^ H9c2 cells, two VDAC1 single guide (sg) RNAs (Fig. 1A: sgRNA 1 and RNA 20) targeting VDAC1 exons 2 and 5, respectively, were designed using the UCSC Genome Browser program (Fig. 1A). The oligonucleotides that included VDAC1 sgRNA 1 or 2 (Fig. 1B, red font], T7 promoter (Fig. 1B, blue font) and CRISPR guide RNA (crRNA, Fig. 1B, green font) sequences were first synthesized and then transcribed into RNA (VDAC1-crRNA) *in vitro* by the T7 RNA synthesis system. Equal molar concentrations of VDAC1-crRNA (fonts in red, blue and green] and CRISPR transacting RNA (tracrRNA, Fig. 1B, light green font, IDT) were first subjected to form the VDAC1-crRNA:tracrRNA duplex by heating at 95°C for 5 min and then cooling to room temperature on the bench top. After that, the VDAC1-crRNA:tracrRNA duplex and Cas9 protein (Fig. 1B: yellow area, purchased from Berkeley University) were mixed to form a ribonucleoprotein (RNP) complex and then transfected into H9c2 cells by the Neon Transfection system. Seventy-two h after transfection, cells were collected after trypsin digest, diluted into 5 cells/ml and then seeded onto 96-well cell culture plates (100 μl/well). After 20 days, the individual cell clones were picked and expanded.

To identify if the individual cell clones were homozygous for VDAC1^−/−^, the cell genomic DNA was extracted and about 300–500 bp length of VDAC1 DNA sequences spanning the sgRNA 1 or 2 sites were PCR amplified with the following primers: sgRNA1: forward VDAC1–5F: 5’-GGGCCTGATGATCGTCTCTG-3’, reverse VDAC1–5R: 5’-GACCGGGAGTTTGGTTCGAA-3’; sgRNA2: forward VDAC1–2F: 5’-TGAGGTTCTGTGCT-GCTCTG-3’, reverse VDAC1–2R: 5’-TGGCTGCTATTCCAAAGCGA-3’. The PCR product was cloned into the TA cloning vector and 5–10 single bacterial colonies were selected, cultured in Lysogeny broth (LB) medium, and the plasmids were extracted for sequencing VDAC1 DNA sequences spanning the sgRNA 1 or 2 area.

### Cell mitochondria, cytosol fraction and western blot analysis

VDAC1^−/−^ and WT H9c2 cells were cultured to confluence in 150 mm dishes and harvested by scrapping. Cells were washed twice with PBS and then resuspended in 0.5 ml of mitochondrial isolation buffer containing (in mM): 200 mannitol, 50 sucrose, 5 KH_2_PO_4_, 5 MOPS, 1 EGTA, and protease inhibitors at pH 7.15. Cells suspended in isolation buffer were homogenized with a Dounce homogenizer and the homogenized slurry was centrifuged for 10 min at 800 *g*. The supernatants were collected and centrifuged for 10 min at 8,000 *g*. After centrifugation, the pellets were designated as the mitochondrial fraction. The supernatants were re-centrifuged at 12,000 *g* for 30 min and the final supernatants were saved and designated as the cytosolic fraction. The final mitochondrial pellets were lysed in RIPA buffer and used for western blot assays.

For western blots, the total cell lysate, mitochondrial and cytosolic protein lysates were resolved by SDS-PAGE. After gel electrophoresis, proteins were transferred onto PVDF membranes. The membranes were incubated with specific primary antibodies: VDAC1 (1:1000, Novrus, Cat: NBP242187); VDAC2 (1:1000, Cell Signaling Technology, Cat: 9412); VDAC3 (1:1000, Proteintech, Cat: 55261–1-Ap); HKII (1:1000, Cell Signaling Technology, Cat: 2867); Bax (1:1000, Cell Signaling Technology, Cat: 14796), COX IV (1:1000, Cell Signaling Technology, Cat: 11967), GAPDH (1:1000, Millipore, Cat: CB1001), Tom-20 (1:1000, Cell Signaling Technology, Cat: 42406) and β-tubulin (1:1000, Cell Signaling Technology, Cat: 2146). Washed membranes were incubated with the appropriate secondary antibody conjugated to HRP and then immersed in an enhanced chemiluminescence detection solution (GE Healthcare) and images were taken using the ChemiDoc imaging system (Bio-Rad).

### Immunofluorescence staining and mitochondrial morphology assessment

Immunofluorescence staining was used to evaluate mitochondrial morphology. Briefly, WT and VDAC1^−/−^ H9c2 cells were fixed with 4% formaldehyde in PBS for 15 min, followed by permeabilization with 100% methanol at −20°C for 10 min, and then incubated with 0.1% Triton X-100 for 15 min at room temperature. The cells were blocked with blocking solution containing 10% goat serum with 2% BSA in PBS for 1 h at room temperature. Primary antibody, anti-COX IV (mouse, Cell Signaling Technology), was diluted in blocking solution and incubated with the cells for 2 h at 37°C. Secondary antibody, anti-mouse conjugated Alexa Fluor 568 (Thermo Fisher Scientific] was diluted in blocking solution and incubated with the cells for 1 h at room temperature. After three more washing with PBS, the cells were stained for nuclei with DAPI (Thermo Fisher Scientific). The coverslips were mounted on glass slides with SHUR/Mount^™^ mounting medium and sealed with nail polish. Single cell images were collected from a Nikon confocal microscope equipped with a 63 × 1.4 NA oil-immersion lens.

Mitochondrial morphology of WT and VDAC1^−/−^ H9c2 cells stained with COX IV were quantified with ImageJ. In brief, the image brightness and contrast were adjusted to make the mitochondria bright and sharp, and then the images’ thresholds were adjusted to give a more accurate representation of the images. After converting the images into binary images, a cell area was selected with the freehand selection tool and then the mitochondria shapes were measured using the tool of analyze particles. Two mitochondrial shape metrics, aspect ratio (AR) and form factor (FF), were calculated [15]. AR represents the ratio of major axis/minor axis of an ellipse fitting to the mitochondrion to reflect length-to-width ratio. FF is considered the perimeter of mitochondria as it is the inverse of circularity to reflect the mitochondrial shape complexity. Both AR and FF have minimal values of 1 when the mitochondrion is a small, perfect circle, and these values increase as the mitochondrion becomes elongated. For each group, 20 cells were analyzed.

### Measurement of ROS production and LDH release

ROS production was assessed by the cytosolic fluorescent ROS indicator, a chloromethyl derivative of 2’,7’ dichlorodihydrofluorescein diacetate (CM-H_2_DCFDA) (Invitrogen) that fluoresces in the presence of H_2_O_2_ and other reactive species including hydroxyl, carbonate and thiol radicals, as well as nitrogen dioxide [16]. LDH release was assessed by the Cytotoxicity Detection Kit (Roche). Briefly, cells were plated into 96-well cell culture plates and cultured in DMEM with 10% FCS and incubated at 37°C with 95% air, 5% CO_2_ for 24 h. Cells were loaded with 5 μM CM-H_2_DCFDA in HBSS for 30 min at 37°C. After removing the loading buffer, the cells were exposed to either 50 or 100 μM tBHP or 50 μM ROT in 4% FCS DMEM followed immediately by baseline fluorescence intensity (F_0_) measurements in a fluorimetric plate reader at 485/530 nm excitation/emission. After F_0_ measurements, the fluorescence intensity of cells was measured every 30 min to assess ROS production. After exposure to tBHP or ROT, the supernatant was collected at 2 and 3 h for LDH release assay as described by the manufacture’s guide. For ROS production, the fluorescence intensities (F) at various times were normalized by subtracting F_0_ from each group respectively. The LDH release was calculated as described by the manufacture’s guide: LDH release = (OD_exp_−OD_con_)/(OD_max_−OD_con_), OD_exp_: the absorbance of treatment cells; OD_con_: the absorbance of no treatment cells; OD_max_: the maximum LDH control activity in the non-treated cells. The maximum LDH control was achieved by lysing cells with a lysis reagent 15 min before collecting the supernatant.

### Cell viability and apoptosis

Cell viability was assessed by the 3-(4,5-dimethylthiazol-2-yl)-2,5-diphenyltetrazolium bromide (MTT) assay as described previously [17]. The MTT assay is a colorimetric assay for assessing cell metabolic activity, which reflects the number of viable cells present. MTT is water-soluble yellow dye that is reduced to its insoluble purple formazan by NAD(P)H-dependent cellular oxidoreductase enzymes in living cells. Formazan is then solubilized with DMSO and the concentration is determined by optical density at 570 nm wavelength. WT and VDAC1^−/−^ H9c2 cells were seeded in 96-well cell culture plates at a density of 0.5×10^4^ cells/well and cultured for 48 h in a 37°C incubator with 95% air, 5% CO_2_. Cells were treated with various concentrations of tBHP for 20 h or ROT for 48 h. After treatment, the cells in each well were incubated with the MTT reagent at a final concentration of 0.5 mg/ml for 3 h at 37°C. After incubation, the culture medium was removed and 100 μl DMSO was added into each well to dissolve the purple precipitate of the MTT inside the cells. The cell plates were placed in a plate reader and absorbance was detected at wavelength 570 nm; cell viability was calculated as the percentage of the absorbance value of control (the value was set to 100 for no tBHP treatment) for the individual cell lines.

To determine if VDAC1^−/−^ affects cell death, the extent of cell apoptosis was determined after exposing cells to the peroxide tBHP. Cell apoptosis was analyzed by TUNEL staining according to the manufacture’s guide (Sigma). WT and VDAC1^−/−^ H9c2 cells were seeded onto glass coverslips in 12-well cell culture plates at a density of 5×10^4^ cells/well and cultured for 48 h at 37°C with 95% air, 5% CO_2_. Cells were treated with various concentrations of tBHP for 20 h. After treatment with tBHP, the cells on coverslips were fixed with 4% formaldehyde in PBS for 1 h, followed by permeabilization with 0.1% Triton X-100 in 0.1% sodium citrate for 2 min on ice. After washing with PBS, the cells were labeled with the TUNEL reaction mixture for 1 h at 37°C in darkness. After labeling, the cells were stained with the nuclear counterstain DAPI and then imaged with the fluorescence microscope with λ_ex_ 505 for TUNEL and λ_ex_ 405 for DAPI. The TUNEL and DAPI stained cells (merged cell) were counted as TUNEL positive cells. The TUNEL positive cells and the DAPI stained cells were counted separately from 5 coverslips for each group. The number of TUNEL positive cells were normalized to the number of DAPI stained cells.

### Mitochondrial mass assay

To determine if VDAC1^−/−^ affects mitochondrial content, the mitochondrial mass was determined by quantification of mitochondrial DNA (mtDNA) using real time PCR. Since H9c2 cells are derived from rat cardiac myoblasts, we designed primers that specifically amplify rat mtDNA. The primers were as follows: forward primer: 5’-CCTTCTCGCTAATTCAGCCT-3’; reverse primer: 5’-GGTTTCGTAATGTTCTCTGGGA-3’. β-actin DNA fragments were amplified as the internal control using the following primers: forward primer: 5’-TGTCCCACCTGTTTGTTCTCT-3’ and reverse primer: 5’-GGTCCTTGGACACCTAGACG-3’. To do the assay, total cell genomic DNA was extracted from one well of cells of the 96-well cell culture plate using DNA extract reagent (Lucigen, QE0905T] and treated at 95°C for 5 min and then at 65°C for 10 min. After treatment, the DNA template concentration was determined using Nanadrop and adjusted to 10 ng/μl. The real time PCR was performed in a total volume of 10 μl containing 5 μl of 2x SYBR Supermix (Bio-Rad), 0.5 μl of forward and reverse primers (400 nM final concentration each], 2 μl of DNA template and 2 μl of water using the following program: 95°C for 3 min for preincubation, and then 95°C for 10 s, 60°C for 60 s for 40 cycles, 65–95°C with 0.5°C increments for the melt curve. The PCR reaction threshold cycle (Ct) value of each DNA sample calculated by the PCR machine software was used to determine the amount of mtDNA and β-actin DNA in each sample. The relative abundance of mitochondrial mass was expressed as the ratio of mtDNA Ct value to β-actin DNA Ct value (mtDNA/β-actinDNA)in the same sample.

### Cell oxygen consumption rate and glycolytic stress

Mitochondrial dysfunction can affect mitochondrial bioenergetics because of defective oxidative phosphorylation, which in turn can lead to altered cellular function. A mitochondrial stress test can be used to measure indices of mitochondrial respiration in intact cells in real time; importantly, it allows for the identification of critical respiratory defects during oxidative stress. In this study, to assess for mitochondrial stress we used a Seahorse XFp analyzer (Agilent) to measure mitochondrial respiration. The mitochondrial stress test was designed to measure key parameters of mitochondrial function by directly measuring the basal O_2_ consumption rate (OCR) of intact cells after the sequential addition of modulators of mitochondrial respiration into the wells containing the cells. The modulators were oligomycin (complex V inhibitor), carbonyl cyanide-4 (trifluoromethoxy) phenylhydrazone (FCCP, a respiratory uncoupler), and ROT and antimycin A (ANT) (complexes I and III inhibitors, respectively). WT and VDAC1^−/−^ H9c2 cells were plated in 100 μl of cell culture medium at 1×10^4^ cells/well in a Seahorse XF Cell Culture Microplate and incubated for 48 h. Cells were treated with DMSO (vehicle] or 125 μM tBHP for 20 h and the OCR was measured according to manufacturer’s instructions. In brief, cells were first washed twice with PBS and then changed to assay medium, i.e. DMEM plus 1 mM pyruvate, 2 mM glutamine, and 10 mM glucose. The cells were incubated at 37°C in a non-CO_2_ incubator for 45 min and then the plate was placed onto the Seahorse XFp analyze. The OCR was recorded over time under basal conditions and in response to 1 μM oligomycin, 1.5 μM FCCP, 1 μM ROT and 1 μM ANT, sequentially. After recording, the cellular protein content in each well was measured by the BCA method and the OCR was normalized to the protein content. Mitochondrial OCR were determined using the Wave program (Agilent). The bioenergetics profiles were obtained as follows: basal respiration, ATP production, H^+^ leak, maximal respiration, spare respiration capacity, and non-mitochondrial O_2_ consumption.

The glycolytic stress test is a standard approach for measuring glycolytic function in intact cells. The cell glycolytic stress is evaluated by directly measuring the ECAR using the Seahorse XFp glycolysis stress test assay. The test determines the ECAR source by sequential injection of glucose, oligomycin and 2-deoxy-glucose (2-DG) into the cell containing wells during the assay. This assay is a reliable approach for determining glycolysis under normal physiological conditions and for compensatory glycolysis following chronic inhibition of mitochondrial ETC complexes. The procedure for measuring ECAR is similar to the measurement of OCR except that the assay medium contains DMEM plus 2 mM glutamine, but no glucose. After recording the basal ECAR, 10 mM glucose, 1 μM oligomycin and 50 mM 2-DG were injected sequentially, and the OCR and ECAR were simultaneously measured. Proton production rate (PPR_tot_) derived from glycolysis (PPR_gly_) and mitochondrial respiration (PPR_resp_) were calculated as described previously [18, 19]. The real time measurements of OCR and ECAR, as indicators of oxidative phosphorylation and glycolysis, respectively, are a convenient way to address a deficit in cellular energy production under abnormal conditions.

### Statistics

All results are expressed as means ± SEM and were analyzed by one-way ANOVA followed by a post-hoc analysis (Student-Newman-Keuls’ test) to determine statistically significant differences in means among groups. A value of p< .05 was considered significant (two-tailed).

## Results

### Generation of VDAC1^−/−^ H9c2 cells by CRISPR-Cas9

VDAC1^−/−^ H9c2 cells were generated using the CRISPR-Cas9 approach. A total of 25 single cell clones for both sgRNAs were expanded and screened for mutations of VDAC1 DNA. Two cell clones, 1B3 and 3B11 were proven to be WT clones as results showed that sgRNA spanning the VDAC1 DNA sequence was the same as for the WT (Fig. 1C). Three clones derived from the sgRNA1, 1G10, 3F2 and 4G10, showed identical mutations among all sequenced DNA clones, for example, 1G10 showed 1bp deletion, 3F2 showed 2 bp inserts and 4G10 showed 1 bp deletion, suggesting the cell DNA has identical mutations in both alleles and indicating they were homozygous mutations (Fig. 1C, D). All other cell clones, 4G10–2, 4F12, etc. showed mixed mutations among the sequenced DNA clones, for example, some DNA clones showed mutation whereas other DNA clones showed WT, indicating the cell DNA had a mutation in one allele, which signifies of heterozygous mutations (Fig. 1C, D).

To further confirm VDAC1^−/−^ in H9c2 cells, the cells were analyzed by western blotting using a specific VDAC1 antibody. Wild-type 1B3 displayed similar level of VDAC1 protein expression as H9c2 cells (Fig. 1E), whereas homozygous DNA mutation cell clones, 1G10, 3F2 and 4G10 exhibited no VDAC1 expression. Interestingly, the heterozygous DNA mutation cell clone 2G11 showed a low level of VDAC1 expression whereas some of the heterozygous DNA mutation cell clones, such as 4F12 and 4G10–2 displayed no VDAC1 expression. These results suggested that cell clones with homozygous mutation were successful in the knockout of the VDAC1 protein in H9c2 cells. Therefore, we used these homozygous DNA mutation cell clones, 1G10, 3F2 and 4G10 for all functional experiments and labelled these clones VDAC1^−/−^C1, VDAC1^−/−^C2 and VDAC1^−/−^C3 H9c2 cells, respectively (Fig. 1C). The sequence alignment of the three rat VDAC isoforms located in the OMM showed 60–70% homology [20]. To explore if expression of VDAC2 or VDAC3 was altered to compensate for VDAC1 knockout, we examined their expression levels in VDAC1^−/−^ C1-C3 (VDAC1^−/−^) H9c2 cells. Both VDAC2 and 3 expression levels were similar in WT and VDAC1^−/−^ H9c2 cells (Fig. 1F), suggesting that VDAC1^−/−^ did not affect expression of VDAC2 and VDAC3.

### Effects of VDAC1^−/−^ on mitochondrial morphology, mass, and bioenergetics

**Fig. 2.**
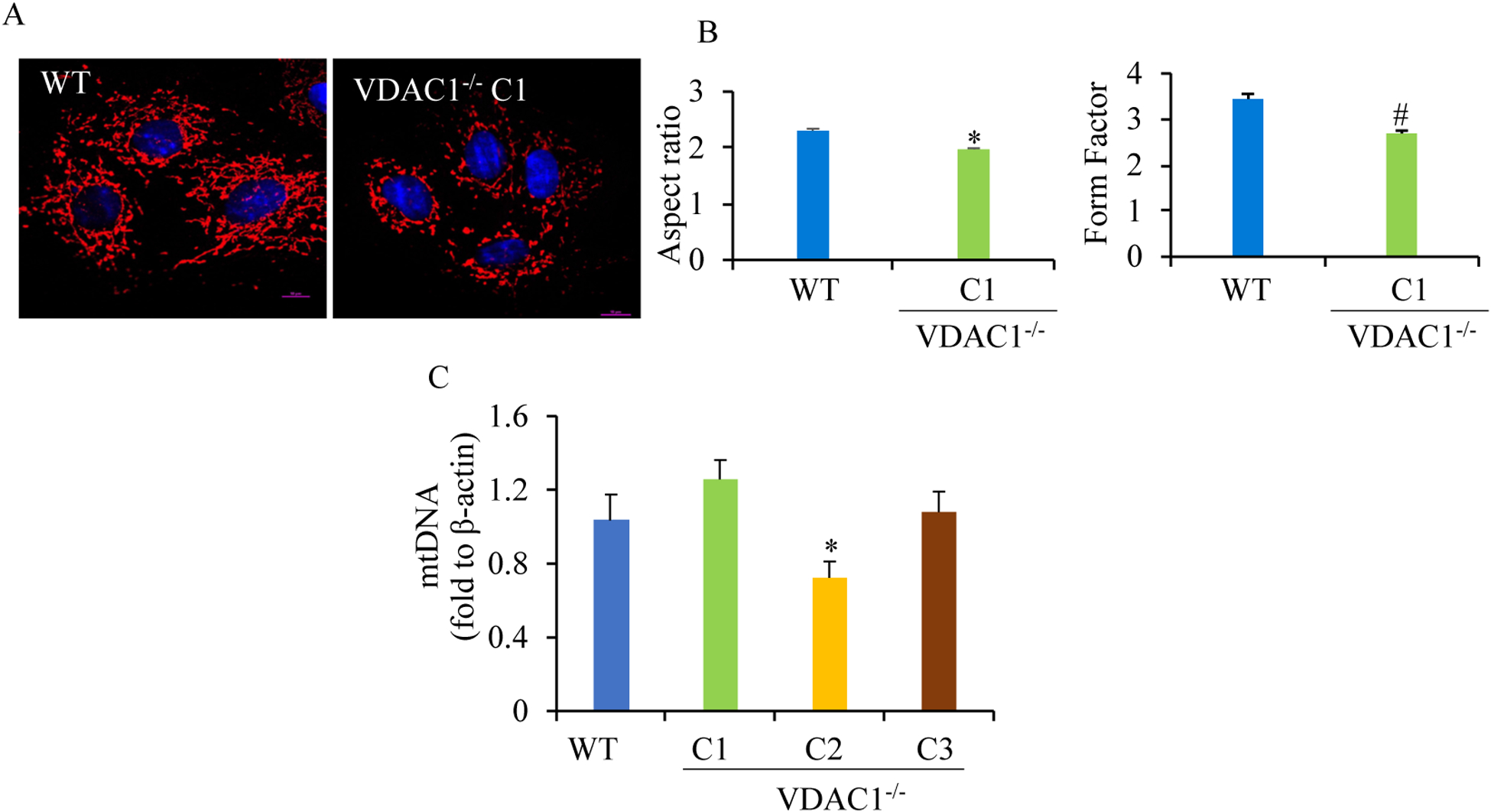
Characteristics of mitochondrial morphology and mass of WT and VDAC1^−/−^ H9c2 cells. (A) Immunostaining of WT and VDAC1^−/−^C1 H9c2 cells with COX IV antibody. (B) Histogram shows average values of two mitochondrial shape metrics, aspect ratio (left panel) and form factor (right panel) from 20 cells/group. * #P<0.05 vs. WT. (C) Histogram shows relative ratio of mtDNA Ct/β-actin DNA Ct detected by real time PCR from WT and VDAC1^−/−^C1, C2 and C3 H9c2 cells. * P<0.05 vs. WT.

After constructing the VDAC1^−/−^ H9c2 cell model, we examined for any alterations in mitochondrial morphology in the VDAC1^−/−^ H9c2 cells. For this purpose, we used VDAC1^−/−^C1 H9c2 cells as a representative clone. WT and VDAC1^−/−^C1 H9c2 cells were subjected to immunofluorescence staining with mitochondrial specific antibody COX IV and examined under confocal microscopy. Fig. 2A showed that WT H9c2 cell mitochondria formed an interconnected filamentous network distributed throughout the cytoplasm; in VDAC1^−/−^C1 H9c2 cells, however, mitochondria aggregated more around the cell nuclei. Analyses of mitochondrial morphology showed that the FF and AR of mitochondria in WT H9c2 cells were significantly greater than in VDAC1^−/−^C1 H9c2 cells (Fig. 2B). These results suggested that VDAC1^−/−^ alters the mitochondrial morphology in H9c2 cells compared to WT cells.

We next investigated if mitochondrial mass was altered in VDAC1^−/−^ H9c2 cells by quantifying the cell mtDNA/β-actinDNA ratio using real time PCR. Results revealed that VDAC1^−/−^C1 and VDAC1^−/−^C3 H9c2 cells had the same mtDNA/β-actin DNA ratio as the WT H9c2 cells (Fig. 2C), whereas the mtDNA/β-actinDNA ratio in VDAC1^−/−^C2 H9c2 cells was lower than in the WT H9c2 cells. The reason for this discrepant result in VDAC1^−/−^C2 H9c2 cells compared to VDAC1^−/−^C1 and C3 H9c2 cells is unclear. Additional experiments, which are outside the scope of this study, should be considered. Nevertheless, the results in VDAC1^−/−^C1 and C3 H9c2 cells suggested that VDAC1^−/−^ did not alter the mitochondrial mass in H9c2 cells.

**Fig. 3.**
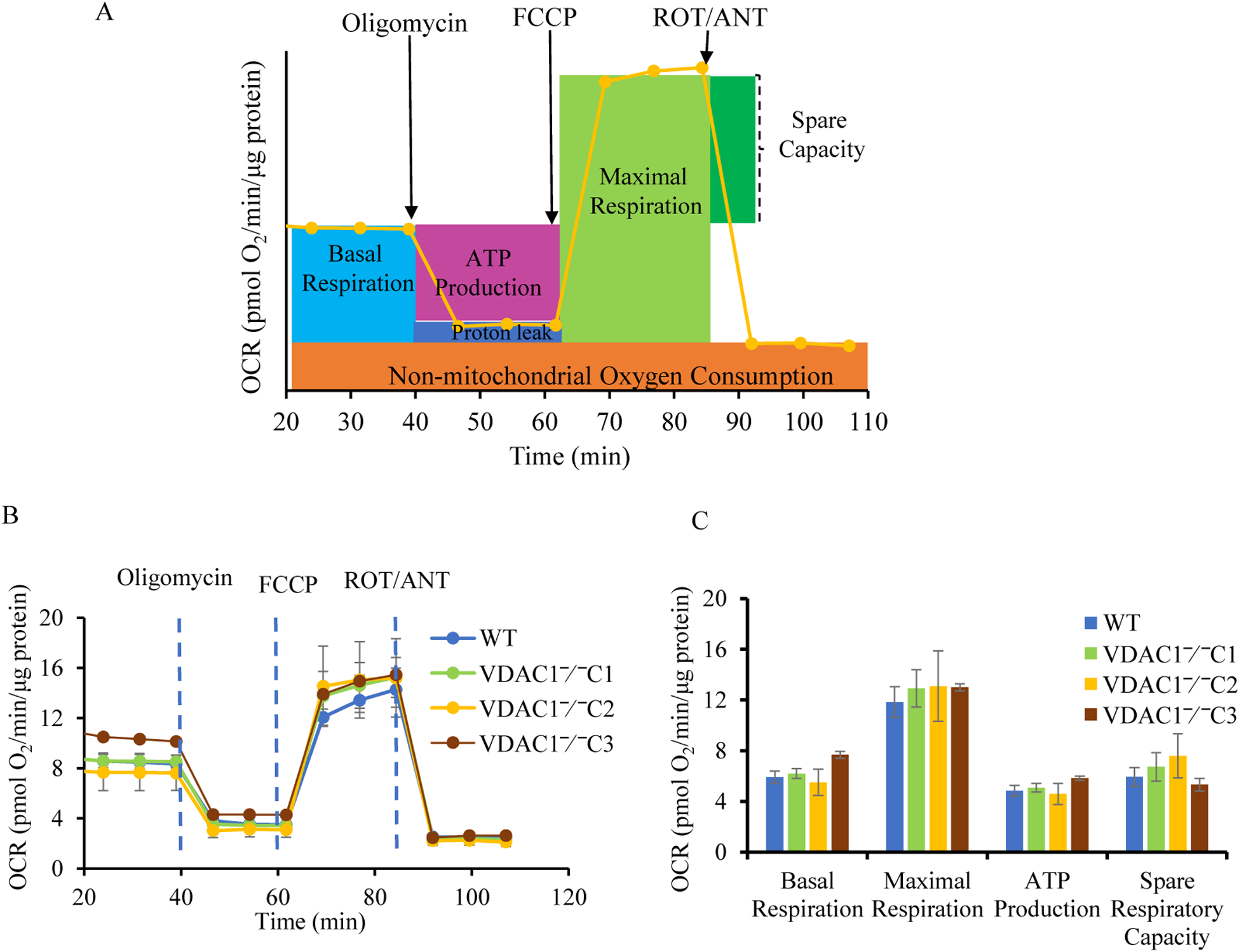
Characteristics of mitochondrial bioenergetics of WT and VDAC1^−/−^ H9c2 cells. (A) Cell mitochondrial stress test profile plot. (B) Traces of O_2_ consumption rate (OCR) in WT and VDAC1^−/−^C1, C2 and C3 H9c2 cells under normal conditions measured using the Seahorse XFp Analyzer. (C) Individual parameters for basal respiration, maximal respiration, ATP production and spare respiratory capacity for WT and VDAC1^−/−^C1, C2 and C3 H9c2 cells. Data are expressed as means ± SEM, n = 8 wells from 2 independent experiments.

To determine if VDAC1^−/−^ alters mitochondrial bioenergetics, OCR in VDAC1^−/−^ and WT H9c2 cells was measured using the Seahorse XFp analyze. The cell mitochondrial stress test profile (Fig. 3A) showed a typical OCR profile under basal conditions and after the sequential addition of oligomycin, FCCP, and ROT/ANT. This sequence allows for assessment and measurement of basal respiration, ATP production, maximal respiration, spare capacity, H^+^ leak, and coupling efficiency. Averaged traces for respiration in WT and VDAC1^−/−^ H9c2 cells are shown (Fig. 3B). As summarized (Fig. 3C), there was no significant difference between VDAC1^−/−^ and WT H9c2 cells in basal respiration, ATP production, maximal respiration, spare capacity, or H^+^ leak. These observations suggest that under basal physiological conditions, VDAC1^−/−^ does not significantly alter mitochondrial respiration.

### VDAC1^−/−^ enhanced tBHP- and ROT-induced cell death independent of mitochondrial bioenergetics

**Fig. 4.**
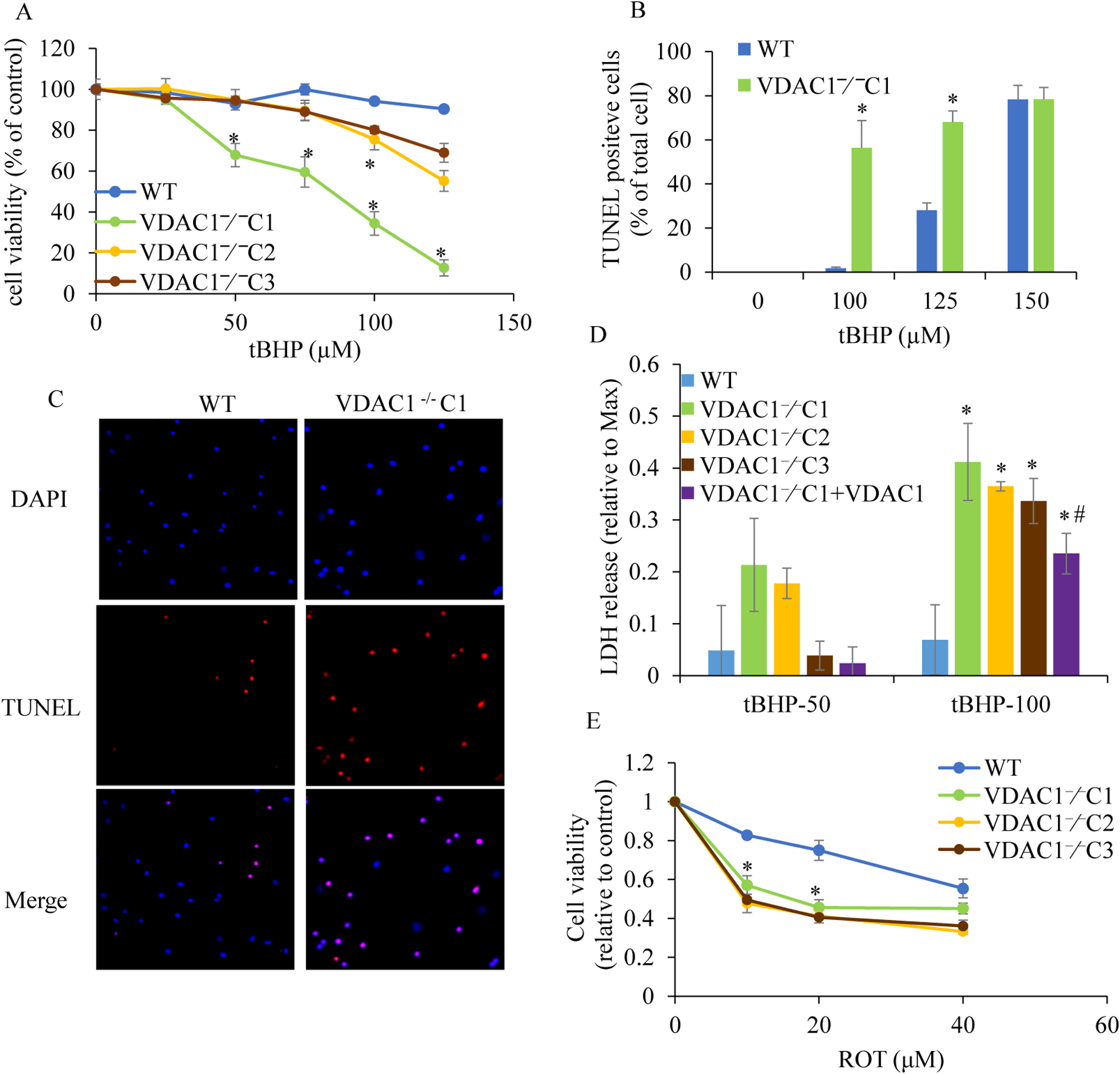
Cell viability and cell death induced by tBHP or ROT in WT and VDAC1^−/−^ H9c2 cells. (A) MTT assay (cell viability) of WT and VDAC1^−/−^ C1, C2 and C3 H9c2 cells after 20 h exposure with various concentrations of tBHP. (B) TUNEL assay of WT and VDAC1^−/−^C1 H9c2 cells after 20 h exposure to various concentrations of tBHP. (C) Representative TUNEL staining (cell death) of WT and VDAC1^−/−^C1 H9c2 cells after 20 h exposure to 125 μM of tBHP. Apoptotic nuclei were TUNEL stained (red) and counterstained with DAPI (blue) to label nuclei. The red and blue merged cells were counted as TUNEL positive cells. (D) LDH release from WT, VDAC1^−/−^C1, C2 and C3 and VDAC1 restored in VDAC1^−/−^C1 H9c2 cells after 3 h exposure to 50, 100 μM tBHP. (E) MTT assay of WT and VDAC1^−/−^C1, C2 and C3 H9c2 cells viability after 48 h exposure to various concentrations of ROT. *P<0.05 vs WT, ^#^ P<0.05 vs VDAC1^−/−^. Each experiment was repeated three times.

We found that under physiological conditions VDAC1^−/−^ caused changes in mitochondrial morphology but no changes in mitochondrial mass (in 2 of 3 VDAC1^−/−^ cell lines) and bioenergetics. We explored, therefore, if VDAC1^−/−^ resulted in changes in mitochondrial bioenergetics, cell viability, apoptosis, and LDH release under oxidative stress conditions. To test this, WT and VDAC1^−/−^ H9c2 cells were incubated with various concentrations of tBHP for 20 h followed by a measure of cell viability using the MTT assay. We found that 94 ± 2% of the WT cells survived after the 100 μM tBHP treatment (Fig. 4A) whereas the viabilities of VDAC1^−/−^C1, C2 and C3 H9c2 cells after a 20 h exposure to 100 μM tBHP were 34 ± 6%, 75 ± 2% and 80 ± 2%, respectively (Fig. 4A), which were significantly lower than that of WT H9c2 cells. In addition, since VDAC1^−/−^C1 appeared to exhibit the greatest vulnerability to tBHP treatment, we determined cell apoptosis in these cells following exposure to 100, 125 and 150 μM tBHP for 20 h. We found that tBHP induced much greater apoptosis in VDAC1^−/−^C1 H9c2 cells than in the WT H9c2 cells (Fig. 4B, C). Exposure to 150 μM tBHP induced greater (higher than 80%) but comparable apoptosis in both VDAC1^−/−^C1 and WT H9c2 cells, suggesting that 150 μM tBHP was near the maximal concentration for inducing full apoptosis of the cells (Fig. 4B). Furthermore, exposure to 50 and 100 μM tBHP caused more LDH release at 3 h, but not at 2 h, in the VDAC1^−/−^C1, C2 and C3 H9c2 cells compared to the WT H9c2 cells; and VDAC1 restoration in the VDAC1^−/−^C1 H9c2 cells significantly reduced LDH release compared to VDAC1^−/−^C1 H9c2 cells (Fig. 4D). Taken together, these results suggested that VDAC1^−/−^ enhances oxidative stress-induced cell death.

tBHP-mediated oxidative stress is caused largely by cytochrome P450 and glutathione peroxidase dependent ROS production in cells [21]. These enzymatic sources of ROS are different from the ROS produced after inhibition of mitochondrial electron transport chain (ETC) complexes (non-enzymatic source). Inhibition of mitochondrial ETC complexes induces formation of the superoxide anion (O_2_^•−^), a progenitor of most ROS [22, 23]. Therefore, we determined if VDAC1^−/−^ affected cell death due to ROS produced by inhibiting the ETC. Cells were exposed to various concentrations of ROT (complex I inhibitor) for 48 h and cell viability was measured by the MTT assay. After exposure to ROT, VDAC1^−/−^C1, C2 and C3 H9c2 cells displayed lower cell viability than the WT H9c2 cells (Fig. 4E), indicating that VDAC1^−/−^ enhances cell death induced by oxidative stress derived from both enzymatic and non-enzymatic sources of ROS. These results additionally suggested that VDAC1^−/−^ H9c2 cells are more vulnerable to oxidative stress than WT H9c2 cells.

**Fig. 5.**
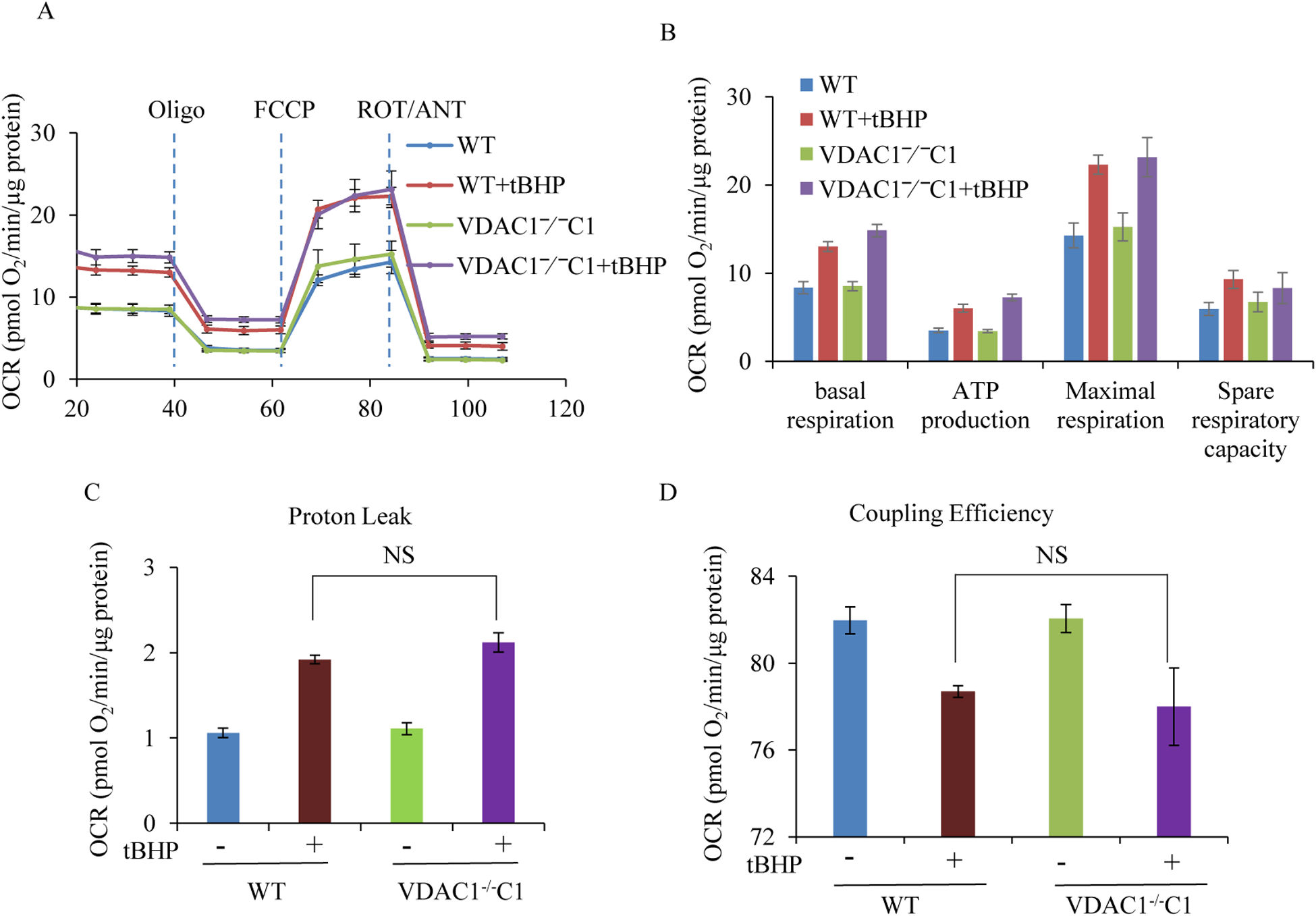
Assay of mitochondrial respiration in WT and VDAC1^−/−^C1 H9c2 cells exposed to tBHP. (A) Traces of O_2_ consumption rate (OCR) in WT and VDAC1^−/−^C1 H9c2 cells with or without exposure to tBHP. (B) Summary of OCR in WT and VDAC1^−/−^C1 H9c2 cells with or without exposure to tBHP. (C) Proton leak and (D) coupling efficiency in WT and VDAC1^−/−^C1 H9c2 cells with or without exposure to tBHP. NS: not significant.

To further explore how VDAC1^−/−^ enhances tBHP-induced cell death, we assessed for mitochondrial stress on WT and VDAC1^−/−^C1 H9c2 cells treated with 125 μM tBHP by measuring OCR, again using the Seahorse XFp analyzer In the absence of tBHP, basal respiration, H^+^ leak, spare respiratory capacity, and ATP production were similar between WT and VDAC1^−/−^C1 H9c2 cells (Fig. 5). In the presence of 125 μM tBHP, in both WT and VDAC1^−/−^C1 H9c2 cells, there was a significant increase in H^+^ leak (Fig. 5C) and a reduction in coupling efficiency (Fig. 5D) of oxidative phosphorylation, which represents maximal ATP synthesis under normal conditions. Moreover, after tBHP exposure, the alteration of mitochondrial respiration was comparable between WT and VDAC1^−/−^ H9c2 cells. These results suggest that the lower viability in VDAC1^−/−^ H9c2 cells compared to WT induced by tBHP was not linked to any change in mitochondrial respiration.

### VDAC1^−/−^ increased ROS production induced by tBHP and mitochondrial complex I inhibition by rotenone

**Fig. 6.**
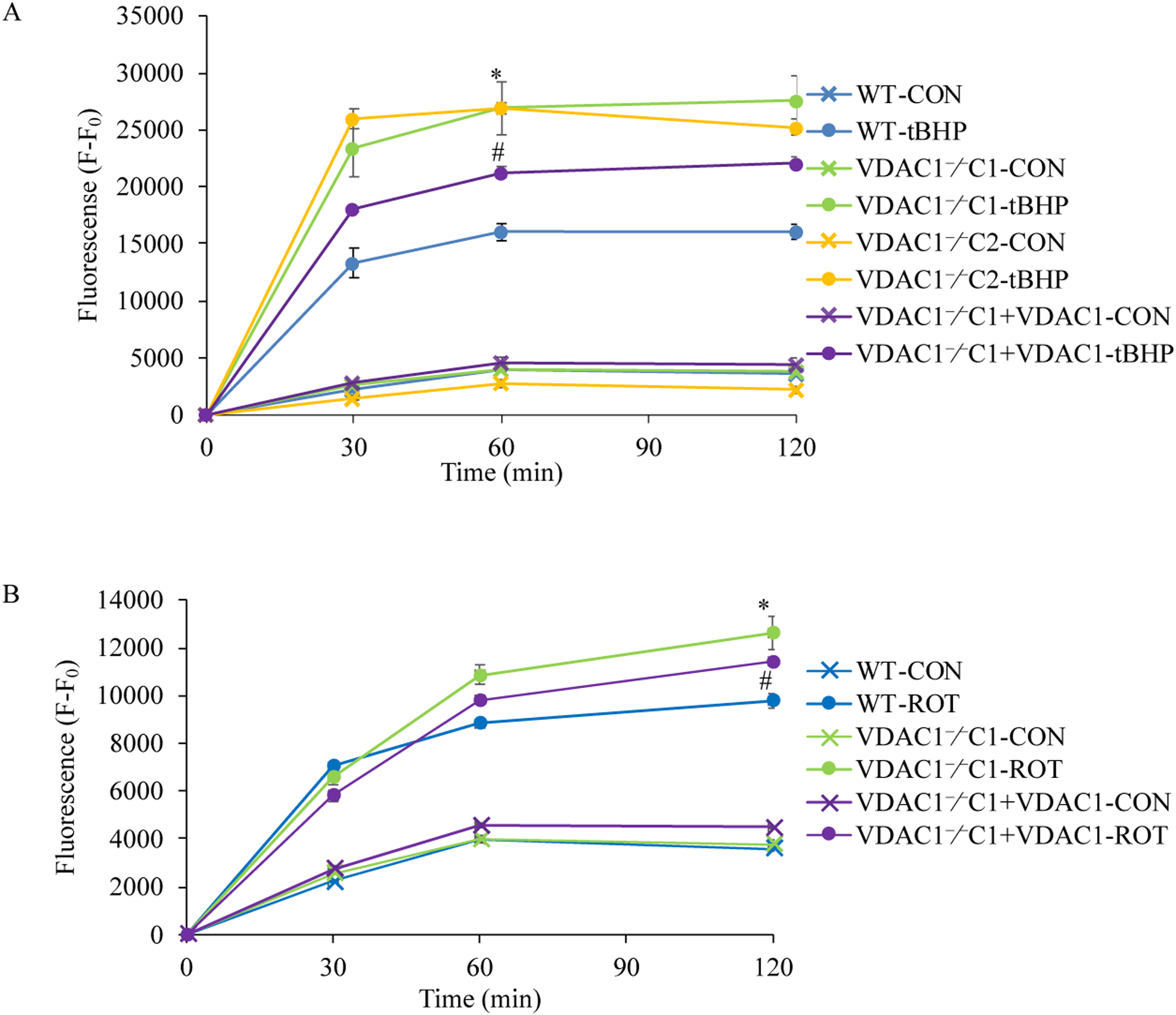
ROS generation in WT and VDAC1^−/−^ and VDAC1 restored VDAC1^−/−^C1 H9c2 cells exposed to tBHP or rotenone with H_2_ DCFDA staining. (A) WT, VDAC1^−/−^ C1, C2 and VDAC1^−/−^ C1+ restored VDAC1 H9c2 cell exposed to 50 μM tBHP. (B) WT, VDAC1^−/−^C1 and VDAC1^−/−^C1+ restored VDAC1 H9c2 cell exposed to 50 μM rotenone. *P<0.05 VS VDAC1^−/−^C1+TBHP. Each experiment was repeated three times.

Previous reports showed that VDAC1 gating modulates mitochondrial metabolism and ROS production [24, 25]. VDAC1^−/−^ enhanced cell death that was induced by ROS originating from both enzymatic and non-enzymatic sources (Fig. 4). Consequently, we determined if VDAC1^−/−^ affected ROS levels produced from these different sources. To test this, VDAC1^−/−^and WT H9c2 cells were loaded with the cellular ROS indicator CM-H_2_DCFDA. After exposure to tBHP or ROT, cellular H_2_DCF fluorescence intensity was determined. Exposure to tBHP increased H_2_DCF fluorescence substantially in VDAC1^−/−^C1, C2 and WT H9c2 cells compared to vehicle-treated cells (Fig. 6A). When compared to WT H9c2 cells, VDAC1^−/−^C1 and C2 displayed greater increases in ROS emission after exposure of tBHP (Fig. 6A), indicating that VDAC1^−/−^ boosted production of cellular ROS induced by tBHP. Furthermore, VDAC1^−/−^C1 H9c2 cells displayed higher ROT-induced ROS production compared to WT H9c2 cells (Fig. 6B). Restoration of VDAC1 in VDAC1^−/−^C1 H9c2 cells significantly decreased ROS emission induced by both tBHP and ROT compared to VDAC1^−/−^C1 H9c2 cells (Fig. 6A, B). These results confirmed that tBHP and ROT induced higher cell death in VDAC1^−/−^ H9c2 cells by stimulating both extra-mitochondrial and mitochondrial sources of ROS, respectively.

### Decreased mitochondria-bound HKII in VDAC1^−/−^H9c2 cells contributed to greater cell death induced by tBHP-mediated oxidative stress

**Fig. 7.**
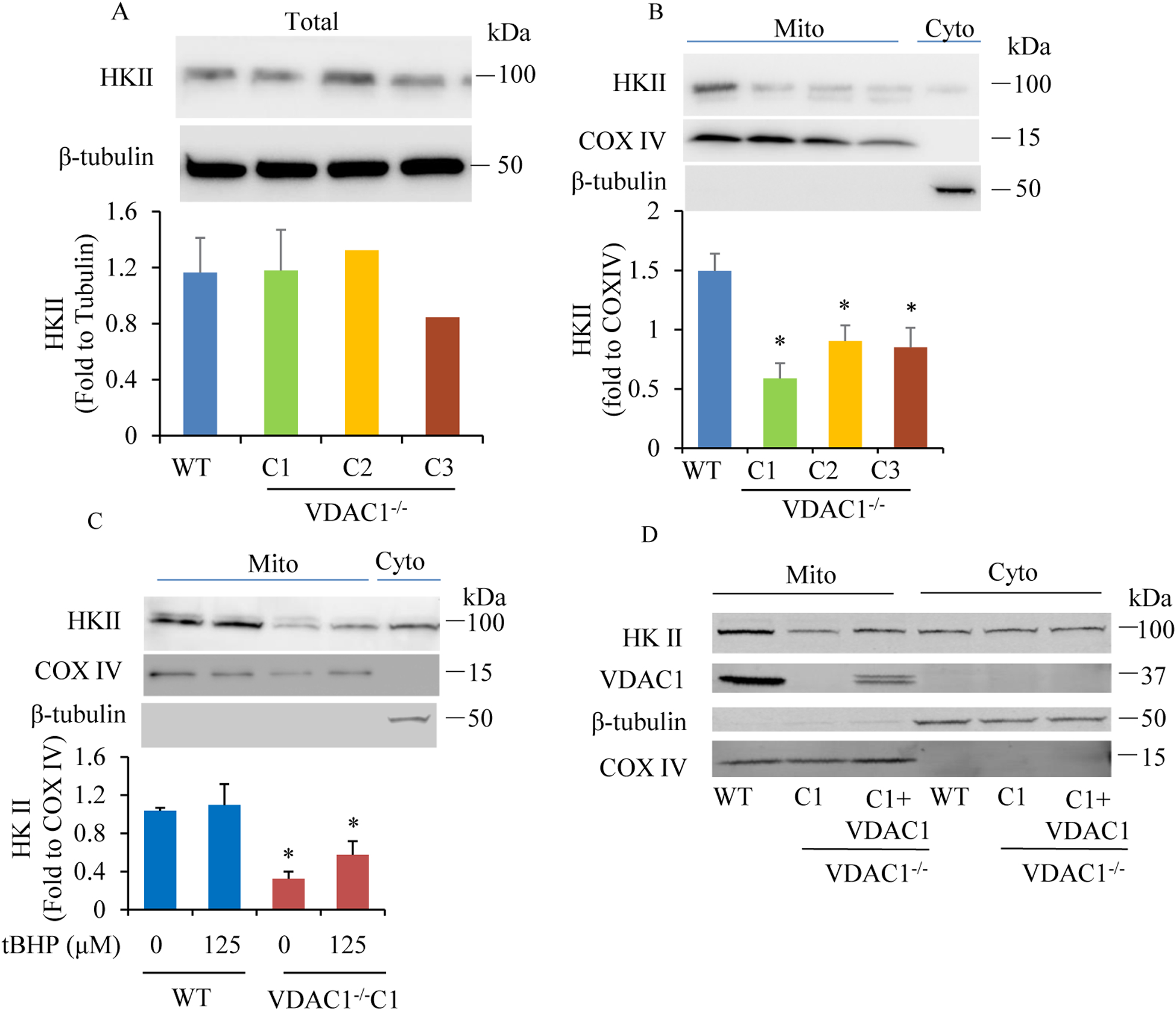
Effect of mitochondria-bound HKII on tBHP-induced cell death in WT and VDAC1^−/−^ H9c2 cells. (A) Western blot analysis of HKII level in WT and VDAC1^−/−^ H9c2 cells from total cell lysate. β-tubulin level was used as protein loading control. (B) Western blot analysis of mitochondrial HKII levels in WT and VDAC1^−/−^H9c2 cells. (C) Western blot analysis of mitochondrial HKII levels in WT and VDAC1^−/−^C1 H9c2 cells exposed to tBHP. (D) Western blot analysis of HKII level in mitochondrial and cytosolic fractions in WT, VDAC1^−/−^ C1 and VDAC1^−/−^C1+ restored VDAC1 H9c2 cells. *P<0.05 vs. WT. Each experiment was repeated three times.

HKII interaction with VDAC1 has been shown to be protective against oxidative stress and cell death [6, 8, 26]. We explored if increased cell death induced by tBHP in VDAC1^−/−^H9c2 cells compared to WT cells could be associated with the mitochondria-bound HKII. HKII expression levels in whole cells and mitochondria in WT and VDAC1^−/−^ H9c2 cells were evaluated. The total HKII levels in WT and VDAC1^−/−^ H9c2 cells were not significantly different under basal (control) conditions (Fig. 7A). However, VDAC1^−/−^ H9c2 cells displayed less HKII levels in the mitochondrial fraction compared to WT H9c2 cells (Fig. 7B). This suggested that VDAC1^−/−^ resulted in less mitochondria-bound HKII. Remarkably, tBHP exposure did not significantly change mitochondria-bound HKII level in both the WT and VDAC1^−/−^ H9c2 cells (Fig. 7C); nevertheless, its level in the VDAC1^−/−^ H9c2 cells was significantly lower than in the WT H9c2 cells. These results suggest that lower levels of mitochondria-bound HKII in the VDAC1^−/−^ H9c2 cells results in greater vulnerability to cell death caused by tBHP oxidative stress.

To further confirm that lower mitochondria-bound HKII in VDAC1^−/−^ H9c2 cells contributed to the greater cell death induced by tBHP, we used the VDAC1^−/−^C1 as the representative clone to restore VDAC1 in VDAC1^−/−^C1 H9c2 cells by stable transfection of VDAC1 WT full-length cDNA into the cells; then we examined mitochondrial HKII level and cell death induced by tBHP. We found that restoration of VDAC1 in VDAC1^−/−^C1 H9c2 cells significantly increased the HKII level in the mitochondrial fraction (Fig. 7D) and rescued the cells from tBHP-induced cell death when compared to VDAC1^−/−^ H9c2 cells (Fig. 4D). These results further confirmed that reduced mitochondria-HKII interaction due to VDAC1^−/−^ contributes to the increase in sensitivity of H9c2 cells to tBHP-mediated cell death via oxidative stress.

### VDAC1^−/−^ H9c2 cells displayed higher extracellular acidification rate derived from glycolysis after exposure to tBHP

**Fig. 8.**
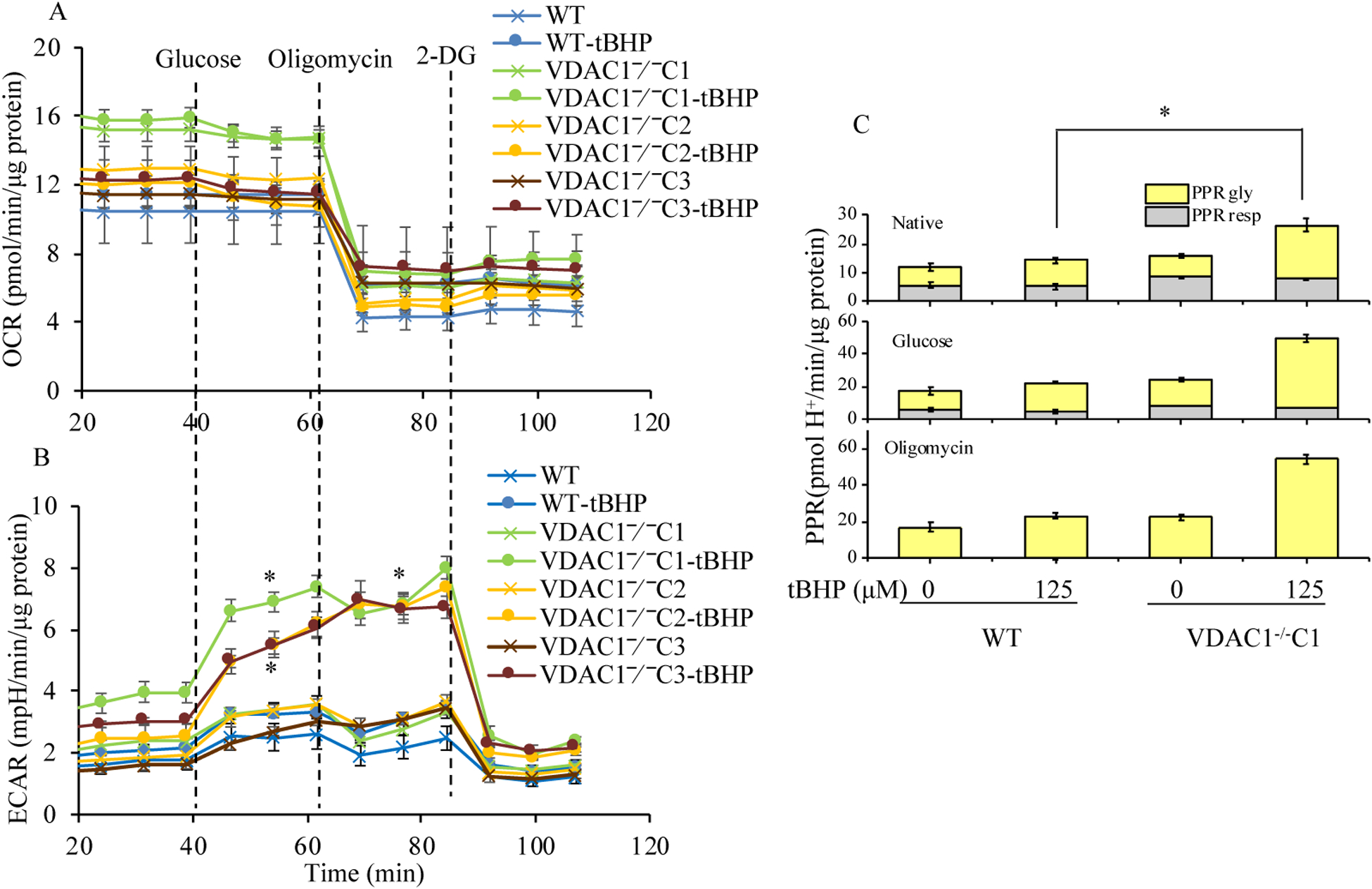
Glycolytic stress in WT and VDAC1^−/−^ H9c2 cells exposed to tBHP. Change in traces of OCR (A) and ECAR (B) in WT and VDAC1^−/−^C1, C2 and C3 H9c2 cells without or with exposure to tBHP for 20 h. Data were expressed as means ± SEM, n = 8 wells from 2 independent experiments. *P<0.05 vs. WT+tBHP. (C) Total proton production rate (PPR_tot_) and the respective contributions of PPR_resp_ and PPR_gly_ before (top panel) and after adding glucose (middle panel) and oligomycin (bottom panel) in WT and VDAC1^−/−^C1 H9c2 cells without or with exposure to tBHP. *P<0.05 WT+tBHP vs. VDAC1^−/−^ +tBHP.

Extracellular acidification arises from glycolytic conversion of glucose, via pyruvate, to lactate coupled with release of H^+^ [18]. There is also hydration of CO_2_ generated from the tricarboxylic acid cycle during aerobic metabolism to form carbonic acid, which dissociates to HCO_3_^−^ and H^+^ in mitochondria [18]. The increased extracellular acidification induces cell death via a ROS- and mPTP opening-mediated mechanism [27]. We examined if VDAC1^−/−^increased tBHP-induced cell death was related to extracellular acidification derived from glycolytic lactate production and/or hydration of mitochondrial derived CO_2_. Under physiological conditions (without tBHP), in both VDAC1^−/−^ and WT H9c2 cells, there was no significant difference in OCR and ECAR after the addition of glucose (Fig. 8A, B). PPR_tot_ (PPR_res_ + PPR_gly_) also did not show any significant difference (Fig. 8C, glucose panel); this suggested that the ECAR was derived from both glycolysis and mitochondrial oxidative phosphorylation. Furthermore, with inhibition of mitochondrial respiration by oligomycin, ECAR levels did not show significant changes compared to the addition of glucose in both the VDAC1^−/−^and WT H9c2 cells (Fig. 8B), but PPR_tot_ was primarily derived from PPR_gly_ (Fig. 8C, oligomycin panel), which suggested that ECAR resulted from increased glycolytic lactate production, after blocking oxidative phosphorylation with oligomycin.

Under stress conditions, i.e. exposure to tBHP, ECAR in VDAC1^−/−^ H9c2 cells was significantly higher than in WT cells and in VDAC1^−/−^ H9c2 cells without tBHP after adding glucose and then oligomycin (Fig. 8B). Moreover, after exposure to tBHP, ECAR in WT H9c2 cells was significantly increased compared to WT without tBHP treatment, but the magnitude of increase was greater in the VDAC1^−/−^ H9c2 cells with tBHP treatment (Fig. 8B). Based on the calculated ECAR produced from PPR_resp_ or PPR_gly_, exposure to tBHP increased PPR, in both VDAC1^−/−^C1 and WT H9c2 cells during baseline and after adding glucose and oligomycin (Fig. 8C, yellow bars). The PPR_gly_ in VDAC1^−/−^C1 H9c2 cells was significantly higher than in the WT cells after treatment with tBHP (Fig. 8C, yellow bars). The PPR_resp_ did not change in both WT and VDAC1^−/−^ H9c2 cells during baseline and after adding glucose (Fig. 8C, grey bars). These results suggested that VDAC1^−/−^ enhanced tBHP- induced higher ECAR, which was derived mostly from glycolysis-related acidification in the VDAC1^−/−^ H9c2 cells.

### VDAC1^−/−^ decreased Bax association with mitochondria

**Fig. 9.**
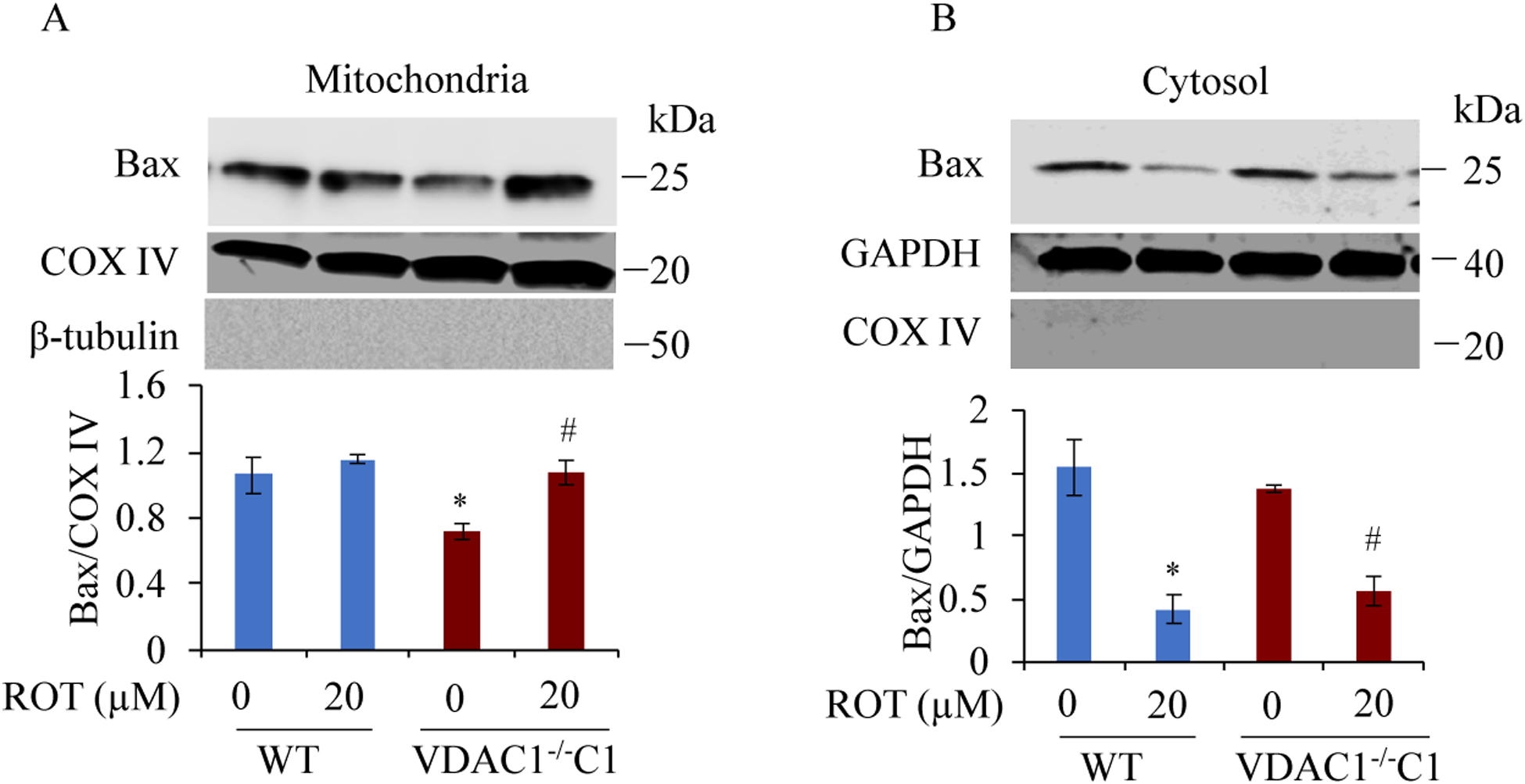
Effect of VDAC l^−/−^ on Bax association with mitochondria. Western blot analyses of Bax levels in mitochondria (A) and cytosol (B) isolated from WT and VDAC1^−/−^C1 H9c2 cells treated with or without 20 μM of ROT for 20 h. COX IV, GAPDH and (β-tubulin were used as protein loading control and as mitochondrial and cytosol markers. *P<0.05 vs. WT non-ROT. #P<0.05 vs. VDAC1^−/−^C1 non-ROT. Each experiment was repeated three times.

We determined if the higher cell death number induced by exposure to ROT in VDAC1^−/−^H9c2 cells compared to WT cells could be related to the pro-apoptotic protein Bax. To test this, we examined Bax content in cytosolic and mitochondrial fractions of VDAC1^−/−^C1 and WT H9c2 cells under control and ROT treatment conditions using western blotting. Fig. 9 shows that in the absence of ROT, the Bax level in the mitochondrial fraction was lower in VDAC1^−/−^C1 H9c2 cells compared to WT cells. In the cytosolic fraction, Bax level was not different between the VDAC1^−/−^C1 cells and WT H9c2 cells. After exposure to ROT, the cytosolic Bax level was decreased to the same degree in both VDAC1^−/−^C1 and WT H9c2 cells when compared to the non-ROT treated cells. The mitochondrial Bax level following treatment with ROT in the VDAC1^−/−^C1 cells was significantly greater when compared to the non-ROT treated VDAC1^−/−^ C1 cells, and it attained the same level of increase as in the WT cells. The mitochondrial Bax level in WT cells showed an increasing trend, but it was not significant when compared to the non-ROT treated WT cells. Our results suggest that VDAC1^−/−^ affects the association of Bax with mitochondria in the control condition, but not under the oxidative stress (ROT) condition.

## Discussion

VDAC1 plays critical roles in energy transduction across the OMM and in apoptotic-mediated cell death modulated by regulatory proteins bound to VDAC [6, 28]. However, the role of VDAC1 in promoting or mitigating cell death remains controversial based on studies using different cell types or cell lines [10, 14]. The role of VDAC1 in promoting or alleviating cell death in cardiac cells and the underlying mechanism have not been critically investigated. To establish its role, we first established a VDAC1^−/−^ H9c2 cell model, then determined that VDAC1^−/−^ altered the incidence of H9c2 cell death induced by oxidative stress, and explored several underlying factors that may explain the role of VDAC1 in cell death. Overall, we found that under physiological conditions, VDAC1^−/−^ and WT H9c2 cells displayed similar cell growth and basal mitochondrial bioenergetics, but with a marked reduction in mitochondria-bound HKII and Bax in VDAC1^−/−^ H9c2 cells. Furthermore, under oxidative stress conditions when cells were exposed to tBHP, VDAC1^−/−^ H9c2 cells showed more apoptosis, greater LDH release, and decreased cell viability than WT cells. Moreover, whereas mitochondrial respiration remained similar in VDAC1^−/−^ and WT H9c2 cells, exposure of VDAC1^−/−^ H9c2 cells to tBHP led to increased ROS production and enhanced ECAR derived primarily from glycolysis-related lactate and H^+^ production. In addition, restoration of VDAC1 in VDAC1^−/−^ H9c2 cells restored mitochondria-bound HKII level, and concomitantly decreased both tBHP-induced ROS production and LDH release. VDAC1^−/−^ H9c2 cells also exhibited greater ROT-induced cell death, which was associated with increased mitochondrial ROS production. The higher cell death of VDAC1^−/−^ cells compared to WT cells induced by ROT exposure does not appear to be mediated via Bax signaling pathway. Taken together, our results showed that VDAC1^−/−^ H9c2 cells had a greater susceptibility to oxidative stress-induced cell death via pathways that are linked to reduced mitochondria-bound HKII, increased ROS production, and enhanced cellular acidification resulting from glycolytic lactate and H^+^ production.

Apoptosis and necrosis are the two major cell death pathways [29, 30]. It remains controversial if VDAC1 mediates or protects against cell apoptosis and necrosis [3]. A recent study [28] reported that VDAC1 oligomerization was induced by apoptotic stimuli and suggested that this promoted cell death by forming a large pore that allowed for release of mitochondrial pro-apoptotic proteins. Brahimi-Horn et al. [14] reported that VDAC1^−/−^ MEFs were more sensitive to apoptotic stimuli than WT MEFs and inferred that VDAC1 protects cells against apoptosis. In contrast, using the same VDAC1^−/−^ MEF model, Baines et al. [10] reported that staurosporine and H_2_O_2_ caused comparable cell death in VDAC1^−/−^ and WT MEFs. They postulated that these agents induce cell death through mPTP pathways and, in so far as VDAC1 is not essential for mPTP opening, it was not involved in the mPTP-driven cell death. In our study we sought to investigate in more detail the role of VDAC1 in oxidative stress-induced cell death by constructing a VDAC1^−/−^ H9c2 cell model. Since we found that VDAC1^−/−^ H9c2 cells exposed to either tBHP or ROT displayed greater LDH release, more apoptosis, and lower cell viability compared to WT (Fig. 4), our results overall suggest that normal expression of VDAC1 in a cardiac cell line protects against oxidative stress induced cell apoptosis and necrosis consistent with the Brahimi-Horn et al. report [14], albeit in a different cell model.

Our results show a decrease in mitochondrial mass in VDAC1^−/−^C2 H9c2 cells (Fig. 2C), but more cell death in VDAC1^−/−^C1 (Fig. 4A), not C2 cells, during t-BHP treatment. This suggests that the VDAC1^−/−^C2 H9c2 cells had a lower mitochondrial content and reduced cell death during tBHP treatment compared to the VDAC1^−/−^C1 H9c2 cells. The reason why less mitochondrial mass does not lead to increased cell death during our stress-imposed condition is not clear. Marquez-Jurado et al. [31] previously reported that in cancer cells, mitochondrial content regulates the expression of apoptotic proteins. They found that cells with higher mitochondrial content contained more cell RNA and higher levels of some apoptotic proteins, which made these cells potentially more vulnerable to die after treatment with apoptotic agents. So, cells with higher mitochondrial content/number can result in greater cell death; this is consistent with our VDAC1^−/−^C1 and C2 H9c2 cells results. However, our results also showed that the VDAC1^−/−^C3 H9c2 cells had similar mitochondrial content as the VDAC1^−/−^C1 H9c2 cells, but had less cell death than the VDAC1^−/−^C1 H9c2 cells which is not compatible with the conclusion that cells with higher mitochondrial content would display greater cell death as reported in cancer cells by Marquez-Jurado et al. [31]. Furthermore, the three VDAC1^−/−^ clones showed similar amounts of LDH release, an index of cell damage, after tBHP exposure and similar cell viability after ROT treatment. Therefore, in our study, mitochondrial content/number may not be the only determining factor for the differential degree of cell death/survival in the different VDAC1^−/−^ H9c2 cell clones induced by tBHP.

To assess potential mechanisms for VDAC1^−/−^ in promoting oxidative stress-induced cell death, we first tested if VDAC1^−/−^ caused changes in mitochondrial respiration. Our results showed that under physiological conditions (Fig. 3, 5), there was no significant difference in cell mitochondrial respiration, suggesting that VDAC1^−/−^ alone did not affect metabolite or ion transfer across the OMM. Expression of VDAC2 and VDAC3 did not change in response to VDAC1^−/−^ in H9c2 cells, so these isoforms could contribute to the normal transfer of substrates to mitochondria in the VDAC1^−/−^ cells. This notion is supported by reports that VDAC1 and VDAC2 co-localize in the same area in the OMM and they both display similar ion selectivity and voltage dependence [6, 32, 33].

When cells were exposed to tBHP, both the VDAC1^−/−^ and WT H9c2 cells displayed comparable increases in basal OCR, higher H^+^ leaks, and lower coupling efficiencies for oxidative phosphorylation, specifically the proportion of O_2_ consumed to ADP phosphorylated for a minimal H^+^ leak (ATP-linked OCR/basal OCR) [34], compared to tBHP untreated VDAC1^−/−^ and WT H9c2 cells (Fig. 5). This observation indicated that compared to WT cells, in VDAC1^−/−^ H9c2 cells, the tBHP-mediated lower cell viability was not mediated by a direct effect of VDAC1 on mitochondrial respiration. In MES cells, it was reported that VDAC1^−/−^reduced O_2_ consumption while mitochondrial coupling efficiency remained unchanged [9]. In MEFs, under normoxic conditions, VDAC1^−/−^ showed lower basal and maximal respiration and ATP synthesis, but H^+^ leak and mitochondrial coupling efficiency were comparable in WT and VDAC1^−/−^ MEFs [14]. Our results showed that under normal conditions, the H^+^ leak rate and mitochondrial coupling efficiency were comparable between WT and VDAC1^−/−^ H9c2 cells, which is consistent with these previous reports [9, 14]. However, our results differ in that we found that basal and maximal respiration and ATP synthesis were not different in WT and VDAC1^−/−^ H9c2 cells, which is not in agreement with prior reports [9, 14]. This may be due to differences in the cell models used in the studies.

To further elucidate the underlying mechanisms, we also addressed if VDAC1^−/−^ affected ROS production in H9c2 cells and if this contributed to enhanced apoptosis and cell death. We found that VDAC1^−/−^ H9c2 cells had a significantly higher ROS production than WT after exposure to tBHP or to ROT (Fig. 6). In addition, the greater ROS production induced by tBHP or ROT in VDAC1^−/−^ H9c2 cells was associated with increased cell death. VDAC1^−/−^ cells had increased ROS production as early as 30 min after adding tBHP or ROT; this was earlier than the LDH release that was evident after 3 h following tBHP exposure, and also earlier than the increases in ECAR and cell apoptosis. Also, the restoration of VDAC1 in VDAC1^−/−^ H9c2 cells decreased tBHP- and ROT-induced ROS production and concomitantly decreased LDH release and increased cell viability. A previous study [24] showed that in cancer cells, VDAC1 opening induced by blocking cytosolic free tubulin binding promoted the flux of respiratory substrates and other metabolites across the OMM. This led to mitochondrial hyperpolarization and increased mitochondrial ROS generation and cell death. However, our results revealed that mitochondrial respiration was similar between VDAC1^−/−^ and WT H9c2 cells in both normal and oxidative stress conditions (Fig. 3, 5); this suggests that VDAC1^−/−^ did not affect substrate and metabolite transfer across the OMM as noted above. These observations further demonstrate that the VDAC1^−/−^-induced increase ROS production was not due to an alteration of mitochondrial respiration as reported by DeHart et al. in their model [24]. Brahimi-Horn et al. reported that VDAC1^−/−^ MEFs produced more H_2_O_2_ than the WT after the cells were cultured for 48 h in normoxic conditions [14]. The authors indicated that the accumulation of ROS in VDAC1^−/−^ MEFs under normoxic conditions was probably due to down-regulation of glutathione peroxidase 7 and the stabilization of hypoxia inducible factor 1α (HIF-1α) [14]. Our results showed that compared to WT, VDAC1^−/−^ enhanced ROS emission only after exposure of cells to tBHP and ROT (Fig. 6). However, under physiological conditions, VDAC1^−/−^ did not induce accumulation of ROS, which was similar to the finding in WT H9c2 cells. Thus, these results are different from those of Brahimi-Horn et al. [14].

The higher cell death in VDAC1^−/−^ H9c2 cells compared to the WT cells after exposure to ROT may be attributed to a role for VDAC1 in controlling the release of mitochondrial superoxide anions (O_2_^•−^) into the cytosol. Under physiological conditions, a net amount of O_2_^•−^is produced (O_2_^•−^emission) as determined by the rate of production minus the rate of scavenging, which keeps the level of O_2_^•−^low [2]. The mitochondrial matrix and intermembrane space are endowed with scavengers that dismutate O_2_^•−^to the non-radical H_2_O_2_. The cytosol also contains antioxidants that neutralize excess O_2_^•−^ exiting mitochondria via VDAC or generated from cytosol. Under oxidative stress, with excess O_2_^•−^ emission, the cellular scavenging system becomes overwhelmed since generation exceeds scavenging [2, 23]. When VDAC is absent or blocked, O_2_^•−^ release from mitochondria could decrease. For example, studies have shown that inhibiting VDAC with DIDS or dextran sulfate significantly reduced mitochondrial O_2_^•−^release in energized mitochondria treated with complex I and III inhibitors, ROT and antimycin A, respectively [35, 36]. This leads to increased mitochondrial O_2_^•− .^ROT drives O_2_^•−^ generation at complex I into the matrix [37]. Knocking out VDAC1, akin to blocking the channel, likely causes accumulation of matrix O_2_^•−^ if it overwhelms the matrix scavenging system. An increase in matrix ROS damages mitochondrial membranes with concomitant release of cytochrome *c* and other pro-apoptotic proteins; this would trigger caspase activation and apoptosis [38]. In the present study, ROT treatment of VDAC1^−/−^ H9c2 cells led to higher ROS production (Fig. 6). We suggest that matrix [O_2_^•−^] was greater because of less release of ROS via VDAC1, which contributes to the higher cell death in these cells.

It is well known that under physiological conditions HKII binds to VDAC1, which blocks direct access of Bax to mitochondria and inhibits cytochrome *c* release and apoptosis [11, 39, 40]. To delineate further how VDAC1^−/−^ increased tBHP-induced cell death, we also examined the status of HKII binding to mitochondria in WT and VDAC1^−/−^ H9c2 cells with or without exposure to tBHP. We showed that VDAC1^−/−^ H9c2 cells displayed low mitochondria-bound HKII levels than the WT H9c2 cells (Fig. 7B), indicating that VDAC1^−/−^ decreases mitochondria-bound HKII. This result is consistent with a previous report [11], which showed that mitochondria-bound HKII activities and expression levels were significantly reduced in heart muscle cells of VDAC1^−/−^ mice. Moreover, we also found that the restoration of VDAC1 in VDAC1^−/−^ H9c2 cells (Fig. 7D) restored HKII binding to mitochondria, which provided compelling evidence that VDAC1, and not VDAC2 or VDAC3, is the predominant mitochondrial docking protein for HKII. This notion is supported by the observation that even though VDAC2 and VDAC3 expressions were evident in our VDAC1^−/−^ cells, they could not compensate for the absence of VDAC1 for HKII binding. Since in VDAC1^−/−^ H9c2 cells the reduced mitochondria-bound HKII did not change with or without exposure to tBHP (Fig. 7C), we explored the impact of the diminished mitochondria-bound HKII in oxidative stress-induced cell death by comparing cell viabilities of VDAC1^−/−^, VDAC1^−/−^ cells with restored VDAC1, and WT H9c2 cells after exposure to tBHP. Our results showed that after exposure to tBHP, VDAC1^−/−^ H9c2 cell viability was lower than in VDAC1^−/−^ cells with restored VDAC1 and WT H9c2 cells. Compared to VDAC1^−/−^ H9c2 cells, restoring VDAC1 in VDAC1^−/−^ H9c2 cells partially increased the mitochondria-HKII interaction and simultaneously decreased tBHP-induced ROS production and cell death (Fig. 4D, 6). Our results suggested that under oxidative stress conditions, diminished mitochondria-bound HKII was, at least in part, responsible for the increase in the VDAC^−/−^ H9c2 cell apoptosis. Previous studies reported that in cancer cells, overexpression of HK by transfection with recombinant HK cDNA, protected cells against apoptotic cell death via increased interaction between HK and VDAC1 [26, 41]. Consistent with these reports and our recent findings [8], we confirmed that the increased interaction between HKII and VDAC1 protects cells against apoptosis. Moreover, we demonstrated that the binding of HKII to VDAC1, rather than the expression level of HKII, was the determining factor in protecting H9c2 cells from oxidative stress-induced apoptosis. Although several studies have shown the impact of HK detachment from mitochondria in cancer and other cell types [42–44], our results show for the first time that decreased mitochondria-bound HKII in VDAC1^−/−^ cells significantly enhances oxidative stress-mediated cell death.

Bax, a pro-apoptotic protein of the BCL-2 family proteins, mediates the intrinsic apoptotic pathway by translocating from the cytosol to mitochondria where it oligomerizes/binds with VDAC and lead to OMM permeabilization with concomitant release of cytochrome *c* [6, 45]. If Bax binds to VDAC1 in mitochondria and if VDAC1 knockout affects the role of Bax in cell apoptosis remains to be determined. Some earlier studies show that Bax interacts with VDAC1 in cerebellar granule neurons and cardiomyocytes under normal conditions, and that anoxia/reoxygenation injury increases Bax binding to VDAC1 [46, 47]. However, recent reports have indicated that VDAC2, not VDAC1, is the mitochondrial receptor for Bax during cell stress and apoptosis induced by BH3-mimetic agents [48, 49]. Our results show that VDAC1^−/−^ reduced the association of Bax with mitochondria under the control condition, but not during ROT mediated oxidative stress, which suggests that VDAC1 can bind with Bax; however, this association is not limited to only VDAC1. Moreover, when compared to non-ROT treated cells, Bax levels increased in the mitochondrial fraction and decreased in cytosolic fraction, respectively, during exposure to ROT in VDAC1^−/−^ H9c2 cells. This suggests that VDAC1^−/−^ does not affect the translocation of Bax under the oxidative stress condition. Lastly, in the VDAC1^−/−^ H9c2 cells, mitochondrial Bax expression level increased to the same level as in the WT cells after exposure to ROT. This result indicates that the higher cell death level in VDAC1^−/−^ cells compared to WT cells does not appear to be mediated via the Bax signaling pathway.

Under physiologic conditions, the major energy generating process in the myocardium is via metabolism of fatty acids (FA) and glucose with a normally greater preference for FA [1]. But under hypoxic or ischemic conditions the heart switches from FA to primarily glucose metabolism [1] so that glycolysis becomes the dominant metabolic pathway [50–52] with much less ATP produced than by oxidative phosphorylation. The increase in glycolytic flux during oxidative stress, e.g. ischemia, contributes to cardiac dysfunction through loss of ionic homeostasis, increased lactate production, and cytosolic acidification (H^+^) due to the uncoupling of glycolysis from oxidative phosphorylation [52–55]. During oxidative stress the increased glycolytic rate to compensate for the diminished ATP production via oxidative phosphorylation is mediated by cytosolic HKII that may not translocate to mitochondria.

Using H9c2 cells, we investigated if VDAC1^−/−^ exacerbated exposure to tBHP-increased ECAR derived from the glycolytic production of lactate and cellular acidification, which might lead to an increase in susceptibility to tBHP-mediated cell death. We simultaneously monitored OCR and ECAR in real time to represent oxidative phosphorylation and glycolysis, respectively, in WT and VDAC1^−/−^ H9c2 cells with or without exposure to tBHP. We found that adding glucose did not change OCR in WT and VDAC1^−/−^ H9c2 cells with or without exposure to tBHP (Fig. 8A). However, adding glucose significantly increased ECAR in VDAC1^−/−^ H9c2 cells exposed to tBHP (Fig. 8B). Further analysis suggests that the tBHP enhancement of ECAR in VDAC1^−/−^ H9c2 cells was due to increased lactate production and cellular acidification, which are major metabolic changes that occur during enhanced glycolysis induced by metabolic stress (Fig. 8C). In excitable cells, accumulation of the glycolytic products lactate and H^+^, during anaerobic metabolism can lead to total loss of ionic regulation and cell death.

Our results also indicated that the oxidative phosphorylation byproduct CO_2_ (Fig. 8C) likely did not contribute to the ECAR. We observed that under oxidative stress, VDAC1^−/−^ increased the uncoupling of glycolysis and oxidative phosphorylation, which led to increased intracellular acidosis and subsequently increased cell death. It is plausible that the VDAC1^−/−^-induced increase of ECAR during oxidative stress is attributed to multiple factors, including less mitochondria-bound HKII and the increase in mitochondria ROS production. We found that VDAC1^−/−^ increased ROS production as early as 30 min after exposure to tBHP or ROT (Fig. 6); this was much earlier than the glycolytic stress-mediated increase of ECAR (Fig. 8). This suggests that the increase in ROS induced by VDAC1^−/−^ with tBHP or ROT exposure initiates the glycolytic stress with an increase in intracellular acidosis that ultimately leads to cell death. Previous studies have shown that detachment of HK from mitochondria caused a decrease in HK activity, an increase in ROS production [56], and potentiated oxidative stress-induced mitochondrial injury and cytotoxicity [44]. Our results are consistent with those studies.

## Conclusion

We generated a VDAC1^−/−^ H9c2 cell model to examine the roles and mechanisms of VDAC1^−/−^ in oxidative stress-induced cell death. Our major new findings are: a) whereas VDAC1 is not required for cell survival in physiologic conditions, it is essential for protection against oxidative stress-induced death in a cardiac cell model; b) VDAC1^−/−^ enhances oxidative stress-induced cell death by decreasing mitochondria-bound HKII with associated increases in ROS production and uncoupling of glycolysis from oxidative phosphorylation, but has no effect on basal mitochondrial respiration; and c) restoration of VDAC1 in VDAC1^−/−^ H9c2 cells reverses the decreased mitochondria-bound HKII in association with decreases in ROS production and cell death. These observations provide novel insights into the critical role of VDAC1. The presence of VDAC1 in OMM during oxidative stress appears to play an essential role in protection against cell death. Furthermore, decreasing ROS production and mitigating cell acidification during oxidative stress support the role of VDAC1 in protecting against cell death. Together, our cell models provide evidence that VDAC1 is a requisite component for protection against oxidative stress-induced cell death, in part, because its absence results in decreased mitochondria-bound HKII associated with increased ROS production and cellular acidification.
